# Superiority of Tumor Location-Modified Lauren Classification System for Gastric Cancer: A Multi-Institutional Validation Analysis

**DOI:** 10.1245/s10434-018-6654-8

**Published:** 2018-07-26

**Authors:** Lin-Yong Zhao, Jun-Jiang Wang, Yong-Liang Zhao, Xin-Zu Chen, Kun Yang, Xiao-Long Chen, Wei-Han Zhang, Kai Liu, Xiao-Hai Song, Jia-Bin Zheng, Zong-Guang Zhou, Pei-Wu Yu, Yong Li, Jian-Kun Hu

**Affiliations:** 1 Department of Gastrointestinal Surgery and Laboratory of Gastric Cancer, State Key Laboratory of Biotherapy, West China Hospital, Sichuan University and Collaborative Innovation Center for Biotherapy, Chengdu, Sichuan Province China; 2 Department of General Surgery Guangdong General Hospital, Guangdong Academy of Medical Sciences, Guangzhou, China; 3 Department of General Surgery and Center of Minimal Invasive Gastrointestinal Surgery, Southwest Hospital, Third Military Medical University, Chongqing, China

## Abstract

**Background:**

The tumor location-modified Lauren classification (mLC) has been proposed recently, but its clinical significance remains under debate. This study aimed to elucidate the clinical relevance of mLC and evaluate its superiority to the Lauren classification (LC) for gastric cancer patients with gastrectomy.

**Methods:**

This study retrospectively evaluated 2764 consecutive gastric cancer patients from three comprehensive medical institutions. The patients were categorized into training, inner-validation, and independent validation sets. The relationships between mLC and other clinicopathologic factors were analyzed, and independent prognostic factors were identified. Survival prognostic discriminatory ability and predictive accuracy were compared between mLC and LC using the concordance index (C-index) and Akaike’s information criterion (AIC), and a nomogram based on mLC was constructed to compare its prognostic improvement with the tumor-node metastasis (TNM) staging system.

**Results:**

A significant association between mLC and gender, age, histologic type, T stage, N stage, and M stage was found. The findings showed that mLC, not LC, is an independent prognostic factor, with a smaller AIC and a higher C-index than LC. The nomogram based on mLC showed a better predictive ability than TNM alone.

**Conclusions:**

Compared with LC, mLC, which could be considered a more reliable prognostic factor, may improve the prognostic discriminatory ability and predictive accuracy for gastric cancer patients with gastrectomy.

**Electronic supplementary material:**

The online version of this article (10.1245/s10434-018-6654-8) contains supplementary material, which is available to authorized users.

As one of the most common gastrointestinal malignances, gastric cancer (GC) is the second leading cause of cancer-related mortality in China, despite a decreasing global incidence.^1^ The prognosis of patients with GC remains poor, and our understanding of this cancer is still limited. The Lauren classification system, which aims to classify GC into the intestinal, diffuse, and mixed types on the basis of histopathologic findings, has been widely used as a pathologic classification system of GC since it was proposed in 1965.^2^ However, the Lauren classification was not correlated with patient survival in some studies,^3^,^4^ perhaps because anatomic and epidemiologic distinctions are not taken into account in the clinical management of GC. Proximal-third or cardia GCs, seldom of a diffuse histologic type, are associated with a worse prognosis than middle- or distal-third GCs,^5^ in which inflammation is associated with chronic gastric acid/bile reflux.^6^ In contrast, distal-third or antral GCs, usually of an intestinal type, often are related to a different type of chronic inflammation (i.e., atrophic gastritis^7^) as a consequence of chronic infection with *Helicobacter pylori,*^8^^,^^9^ which may be the driving force for carcinogenesis.

Gastric cancers of the diffuse type are particularly lacking inflammation in the presence a germline mutation in CDH1.^10^ Hence, Shah et al.^11^ advocated that the Lauren classification be modified to include both the Lauren histopathologic classification and the anatomic location of GC, yielding at least three entirely distinct types, namely, the proximal non-diffuse type (PND), the distal non-diffuse type (DND), and Lauren’s diffuse type (D). Then they showed that differentially expressed genes could distinguish the GC subtypes from each other and from adjacent normal gastric mucosa.

Nevertheless, no unanimous consensus has been reached on whether the modified Lauren classification is superior to the Lauren classification, and the clinical significance of the modified Lauren classification has not been well defined. Therefore, it is necessary to clarify the clinical significance of the modified Lauren classification and to compare it with the Lauren classification for GC patients.

In the current study, we evaluated the clinical relevance and prognostic significance of the modified Lauren classification and assessed whether it can improve prognostic discriminatory ability and predictive accuracy for patients with GC enrolled at three different medical institutions.

## Patients and Methods

### Patients

Patient records were rendered anonymous and de-identified before analysis, and signed informed consent was waived due to the retrospective nature of the analysis. This retrospective study was approved by the Surgical Gastric Cancer Patient Registry (no. WCH-SGCPR-2018-06).

**Fig. 1:**
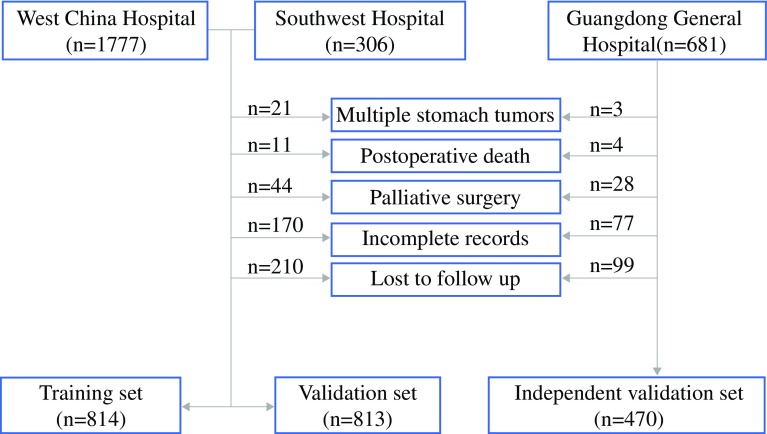
The flow diagram of patients enrolled in this study

The study retrospectively evaluated 2764 consecutive GC patients who received gastrectomy in West China Hospital, Guangdong General Hospital, or Southwest Hospital from January 2008 to June 2014. The diagnosis of primary GC for all the patients was confirmed by upper gastrointestinal endoscopy and biopsy. The exclusion criteria ruled out multiple stomach tumors, death due to postoperative complications in the hospital, palliative surgery, incomplete medical records, and loss to follow-up evaluation. Finally, 2097 patients were enrolled in this study according to the flow diagram in Fig. 1. The 1627 patients enrolled from West China Hospital and Southwest Hospital were randomly divided into a training set and an inner validation set, with a sample size ratio of 1:1 using X-tile,^12^ whereas the 470 patients from Guangdong General Hospital were defined as an independent validation set.

### Follow-up Evaluation and Survival

After undergoing gastrectomy, all the patients were periodically followed up by outpatient visits and telephone interviews. The follow-up evaluation was every 3 months during the first 2 postoperative years, every 6 months in the third year, and annually thereafter until death. Overall survival (OS) was the primary end point, and the survival time was calculated from the date of operation to the date of death or the last follow-up visit, in June 2017. Of the 2764 patients, 2455 (88.8%) were followed up.

### Definition of the Modified Lauren Classification

According to Lauren’s criteria, GC could be histopathologically categorized into three subtypes (intestinal, diffuse and mixed type) called the Lauren classification. In this study, the Lauren classification was modified by the combination of tumor location, resulting in the tumor location-modified Lauren classification (mLC), which included the proximal non-diffuse (PND), diffuse (D), and distal non-diffuse (DND) types.

Specifically, the PND tumors were those whose bulk (> 80%) was located in the gastric cardia, with the Lauren intestinal or mixed type shown on histopathology, and could extend up to the gastroesophageal junction and a small portion of the distal esophagus. The D tumors could be located anywhere in the stomach, with the Lauren diffuse type shown on histopathology, whereas the DND tumors usually were located in the distal stomach and could extend up to the middle body of the stomach or down to the pylorus, with the Lauren intestinal or mixed type shown on histopathology.^11^

The patients were classified according to the tumor location and Lauren classification based on the final pathologic report. The tumor-node-metastasis (TNM) stage was evaluated according to the seventh edition of the American Joint Committee on Cancer (AJCC) TNM staging system.^13^

### Statistical Analysis

All the statistical analyses were performed using R program, version 3.1.2 (URL http://www.R-project.org/.) and SPSS, version 23.0 (SPSS, Inc., Chicago, IL, USA) as well as GraphPad Prism 5 (GraphPad Software Inc., San Diego, CA, USA). Logistic regression analysis was used for multicollinearity analysis and to identify the risk factors for mLC, whereas Cox’s proportional hazard regression model, with the conditional backward stepwise procedure, was used in the uni- and multivariate survival analyses. The cumulative survival rates were calculated using the Kaplan-Meier method, with subgroups compared by the log-rank test.

The nomogram was displayed with the package of Regression Modeling Strategies (URL http://CRAN.R-project.org/package=rms) in R. The predictive accuracy of the nomogram then was validated using a calibration curve and receiver operating characteristic curve (ROC) quantified by the area under the curve (AUC). An AUC of 1.0 shows perfect concordance, whereas an AUC of 0.5 indicates no relationship.^14^

The Harrell Miscellanceous package (URL http://CRAN.R-project.org/package=Hmisc.) was used to illustrate the comparison of the prognostic prediction, in which the concordance index (C-index) and Akaike’s information criterion (AIC) values within a Cox proportional hazard regression model were calculated for a survival prediction system to show the accuracy and discriminatory ability, respectively. A smaller AIC value indicated a better model for predicting outcome, whereas a larger C-index demonstrated a more accurate prognostic prediction.^15^^,^^16^ A *p* value lower than 0.05 (two-sided) was defined to be statistically significant.

## Results

### Correlation Analysis Comparing Clinicopathologic Factors and mLC

Table S1 compares the clinicopathologic factors between the training and validation sets, and between the three modified Lauren classification (mLC) subtypes. No significant difference was found between the training and validation sets in terms of any clinicopathologic factors (all *p* > 0.05), illustrating that the baseline was balanced between them. Moreover, both in the training set and the validation set, the mLC subtypes were found to be significantly associated with gender, age, histologic type, T stage, N stage, and M stage (all *p* < 0.05). Specifically, patients with the D (diffuse) type were more likely to be female and younger than 60 years with the histologic type of the signet ring cell, deeper tumor invasion, wider lymph node metastasis, and distant metastasis. However, mLC was not significantly associated with macroscopic type (*P*_1_ = 0.070; *P*_2_ = 0.348), tumor size (*P*_1_ = 0.148; *P*_2_ = 0.063), or chemotherapy status (*P*_1_ = 0.075; *P*_2_ = 0.093).

### Identification of Risk Factors and Multicollinearity Analysis for mLC

As demonstrated in Table S2, logistic regression analyses were performed to evaluate multicollinearity and to determine the risk factors for mLC. In the univariate analysis, the involved factors consisted of the clinicopathologic factors, gender, age, histologic type, T stage, N stage, and M stage. However, multivariate logistic regression model analysis indicated that only histologic type (odds ratio [OR], 1.032; 95% confidence interval [CI], 1.042–1.449) and M stage (OR, 1.305; 95% CI, 1.202–1.616) were independent risk factors for mLC.

### Uni- and Multivariate Analyses of Factors Associated With Prognosis

**Fig. 2:**
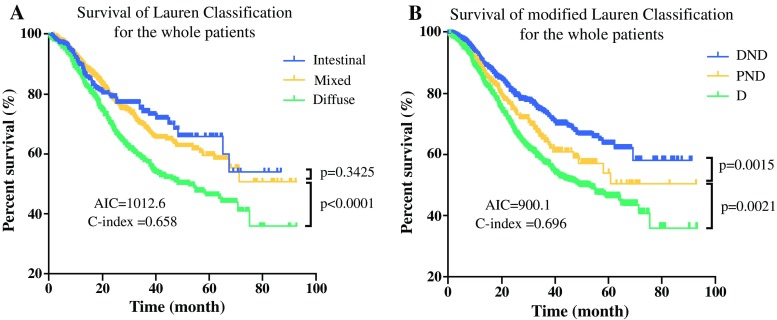
Kaplan-Meier survival analysis of the Lauren classification (**a**) and the modified Lauren classification system (**b**) for the whole cohort. The significance of the difference between survival curves was calculated by the log-rank test

The 3- and 5-year overall survival (OS) rates for different subtypes in mLC are shown in Table S3. The 3-year OS was 51.2% for D, 60.7% for PND, and 67.0% for DND, whereas the corresponding 5-year OS rates were 41.9%, 53.8%, and 54.2%. The multivariate analysis presented in Table S4 shows that the independent prognostic factors for GC patients were mLC (hazard ratio [HR], 1.116; 95% CI, 1.018–1.435; *p* = 0.039), T stage (HR, 1.404; 95% CI, 1.091–1.897; *p* = 0.012), N stage (HR, 1.612; 95% CI, 1.127–1.991; *p* < 0.001), and M stage (HR, 1.917; 95% CI, 1.622–2.361; *p* < 0.001), but not LC (*p* = 0.082). Furthermore, Kaplan-Meier survival analysis demonstrated that the LC system and the mLC system could be regarded as prognostic factors (*p* < 0.05; Table S3; Fig. 2).

### Comparison and Validation of mLC With LC

**Fig. 3:**
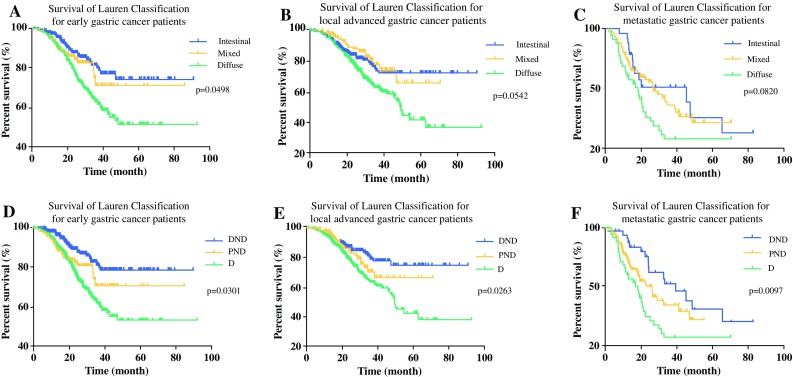
Comparison of the predictive ability between the Lauren classification and modified Lauren classification systems for early gastric cancer, local advanced gastric cancer and metastatic gastric cancer

The C-index values and AIC for different systems were calculated to compare their predictive accuracy and prognostic discriminatory ability, respectively (Table S3). Compared with the LC system (AIC, 1012.6; C-index, 0.658), the mLC system (AIC, 900.1; C-index, 0.696) had a larger C-index and a smaller AIC value (*p* < 0.05; Fig. 2), suggesting that the mLC system was better than the LC system in terms of survival predictive accuracy and discriminatory ability for the whole cohort of patients. Moreover, overlapping curves were found in the LC system but not in the mLC system for local advanced GC and metastatic GC (Fig. 3), respectively, with no significant difference in survival rate between the intestinal and mixed subtypes in the LC system, highlighting that mLC had better discriminatory ability and more predictive accuracy than LC in prognostic prediction, especially for local advanced and metastatic GC. For early GC, mLC also showed better discriminatory ability than LC, although overlapping curves existed in both systems (Fig. 3).

**Fig. 4:**
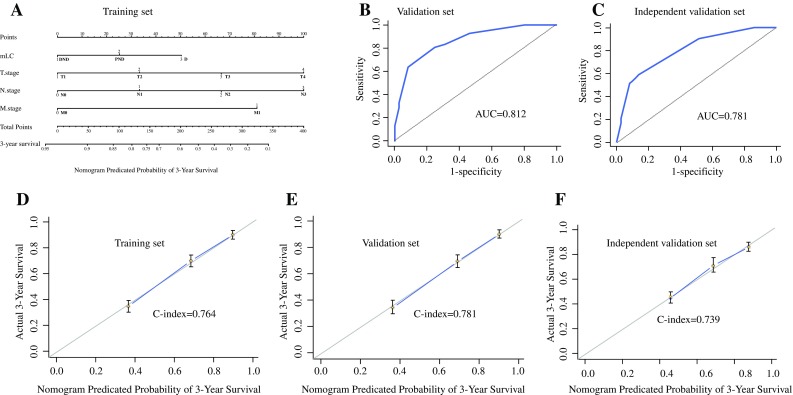
**a** Nomogram composed of the independent prognostic factors to predict the 3-year overall survival rate of gastric cancer patients, while **b** and **c** show ROC curves after applying this nomogram in the inner validation and independent validation sets and calibration curves (**d**, **e**, **f**) produced in the training, inner validation and independent validation sets, respectively, after applying this nomogram. The risk value of the 3-year overall survival rate was calculated by drawing a vertical line to the point on the axis for each of the factors. The points for each factor are summed and given to the point line. Thereafter, the bottom line corresponding vertically to the above total line demonstrates the individual predictive value for the 3-year overall survival rate

A nomogram was constructed to predict the 3-year OS for patients in the inner training set (Fig. 4A), and independent prognostic factors, such as the mLC system, T stage, N stage, and M stage, were enrolled in the nomogram plots, which were similar to those in the aforementioned multivariate analysis by Cox regression. The calibration curve to validate this nomogram in the training set is displayed in Fig. 4D (C-index, 0.764), demonstrating that the nomogram based on mLC showed better predictive ability than TNM alone (C-index, 0.721; Table S3). The predictive probability of 3-year survival was closely similar to the actual 3-year survival when the nomogram based on the mLC system was applied.

Additionally, we applied this nomogram in the inner validation set and the independent validation set using calibration curves and ROC curves, and the corresponding calibration curve (C-index, 0.781 vs 0.739) and ROC curve (AUC, 0.812 vs 0.781) in the two sets suggested a good predictive ability for 3-year survival (Fig. 4).

## Discussion

Since Lauren proposed a histopathologic classification of GC into two distinct types, the intestinal type and the diffuse type,^2^ several studies^17^^–^19 have demonstrated that the diffuse type is most frequently seen in women and younger patients, consistent with what we found in the current study. Moreover, the tumor mLC was significantly associated with T stage, N stage, and M stage. Specifically, the diffuse type also was associated with deeper tumor invasion, wider lymph node metastasis, and distant metastasis in the current study, indicating that patients with the diffuse type were more likely to present worse biologic behavior and more aggressive features than those with non-diffuse types, which could be the reason why the diffuse type also is frequently seen in younger patients, because GC tends to exhibit more aggressive tumor behavior in young patients than in old patients.^20^

Moreover, mLC was compared with the traditional LC. In the current study, the mLC system was better than the LC system in terms of survival predictive accuracy and discriminatory ability, illustrating that intestinal and mixed types combined with tumor location could predict survival more accurately than without locations because the mLC and LC differed essentially in tumor locations, with intestinal and mixed type classified by LC. Patients with proximal GC usually have a worse prognosis than those with distal tumor, and the cause for the proximal inflammation usually involves chronic gastric-acid/bile reflux, whereas the chronic infection with *Helicobacter pylori* may promote carcinogenesis.

The mLC system, together with T, N, and M stages, was an independent prognostic factor, whereas the LC system was not. In a previous Korean study,^21^ the mLC system was demonstrated to be an independent prognostic factor only for early GC, whereas our results suggested that it is a prognostic factor not only for early GC, but also for local advanced and metastatic GC. This discrepancy might be because the proportions of early GC in Korean and China are different: Approximately 60% of Korean patients have GC diagnosed in its early stages, whereas among Chinese patients, the proportion of early cancers is much lower (15.4%) and the advanced stages are more frequent.^22^

Additionally, a good staging system, which would be of great value for the management of GC, should be able to distinguish the survival curves between several subgroups of patients, and to provide accurate prognostic estimation and guidance for choosing the appropriate adjuvant treatment.^23^ Therefore, we used the C-index and AIC to show the improvement in prognostic prediction. The mLC system had a higher C-index and a smaller AIC value than the LC system, suggesting that the mLC system is better than the LC system in terms of survival predictive accuracy and discriminatory ability. Meanwhile, the nomogram constructed to predict 3-year overall survival also was applied in our study to demonstrate the prognostic significance of independent factors for GC patients. The high predictive accuracy of the nomogram based on mLC was demonstrated through calibration curves and ROC curves in the validation set and independent validation set. This nomogram based on the mLC system showed a more powerful survival discrimination and a better predictive accuracy than that of TNM staging system alone for GC patients with gastrectomy.

Our study also had some limitations. For example, although it was a non-randomized retrospective multi-institutional study, some parameters could have been observed by chance in spite of the large sample. Additionally, as an observational study, selection bias also existed, and about 24% of the entire data set was excluded, mainly because of the incomplete records and follow-up information. Therefore, large-scale and prospective multicenter studies are needed to elucidate the clinical relevance of mLC for GC before stronger conclusions can be drawn.

## Conclusion

For gastric cancer patients with gastrectomy, the mLC system, which could be considered a reliable prognostic factor, had better prognostic discriminatory ability and accuracy than the LC system.

## Electronic supplementary material

Below is the link to the electronic supplementary material.

Supplementary material 1 (DOCX 33 kb)

Supplementary material 2 (DOCX 20 kb)

Supplementary material 3 (DOCX 24 kb)

Supplementary material 4 (DOCX 49 kb)
