# Supplementary material for: Superiority of Tumor Location-Modified Lauren Classification System for Gastric Cancer: A Multi-Institutional Validation Analysis
Source: Ann Surg Oncol. 2018 Jul 26;25(11):3257–63. doi: 10.1245/s10434-018-6654-8 (PMC6132412; doi:10.1245/s10434-018-6654-8)
Supplement: Supplementary file 1 — Supplementary material 1 (DOCX 33 kb) [file 10434_2018_6654_MOESM1_ESM.docx]

| Table s1. Demographic and clinicopathologic characteristics. | | | | | | | | | | | | |
| --- | --- | --- | --- | --- | --- | --- | --- | --- | --- | --- | --- | --- |
|  | **Training set** | | | | |  | **Validation set** | | | | | P***** |
| Factors | PND  (n=149) | DND (n=279) | D  (n=386) | Total  (n=814) | P_1_ | | PND  (n=128) | DND (n=281) | D  (n=404) | Total  (n=813) | P_2_ |  |
| Gender |  |  |  |  | <0.001 | |  |  |  |  | 0.023 | 0.138 |
| Male | 119 | 204 | 173 | 496 |  | | 101 | 196 | 137 | 434 |  |  |
| Female | 30 | 75 | 213 | 318 |  | | 27 | 85 | 267 | 379 |  |  |
| Age (years) |  |  |  |  | <0.001 | |  |  |  |  | 0.001 | 0.262 |
| ≥60 | 91 | 146 | 144 | 381 |  | | 68 | 138 | 152 | 358 |  |  |
| <60 | 58 | 133 | 242 | 433 |  | | 60 | 143 | 252 | 455 |  |  |
| Macroscopic type |  |  |  |  | 0.070 | |  |  |  |  | 0.348 | 0.085 |
| Borrmann 0-II | 80 | 164 | 192 | 436 |  | | 73 | 168 | 219 | 470 |  |  |
| BorrmannIII-IV | 69 | 115 | 194 | 378 |  | | 55 | 113 | 185 | 343 |  |  |
| Histologic type |  |  |  |  | <0.001 | |  |  |  |  | <0.001 | 0.085 |
| Well/Moderately | 63 | 105 | 57 | 225 |  | | 57 | 127 | 66 | 250 |  |  |
| Poorly/Undifferentiated | 57 | 103 | 188 | 348 |  | | 43 | 93 | 168 | 304 |  |  |
| Signet ring cell | 29 | 71 | 141 | 241 |  | | 28 | 61 | 170 | 259 |  |  |
| Tumor size |  |  |  |  | 0.148 | |  |  |  |  | 0.063 | 0.084 |
| ≤5cm | 77 | 164 | 199 | 440 |  | | 74 | 179 | 221 | 474 |  |  |
| >5cm | 72 | 115 | 187 | 374 |  | | 54 | 102 | 183 | 339 |  |  |
| T Stage |  |  |  |  | <0.001 | |  |  |  |  | <0.001 | 0.110 |
| T1-2 | 37 | 128 | 88 | 253 |  | | 29 | 148 | 106 | 283 |  |  |
| T3-4 | 112 | 151 | 298 | 561 |  | | 99 | 133 | 298 | 530 |  |  |
| N Stage |  |  |  |  | <0.001 | |  |  |  |  | <0.001 | 0.094 |
| N0 | 49 | 113 | 77 | 239 |  | | 46 | 107 | 82 | 270 |  |  |
| N1-3 | 100 | 166 | 309 | 575 |  | | 82 | 174 | 322 | 543 |  |  |
| M Stage |  |  |  |  | <0.001 | |  |  |  |  | <0.001 | 0.101 |
| M0(Early) | 17 | 78 | 54 | 149 |  | | 20 | 108 | 55 | 183 |  |  |
| M0(Local advanced) | 118 | 175 | 277 | 570 |  | | 93 | 153 | 289 | 535 |  |  |
| M1 | 14 | 26 | 55 | 95 |  | | 15 | 20 | 60 | 95 |  |  |
| Chemotherapy |  |  |  |  | 0.075 | |  |  |  |  | 0.093 | 0.364 |
| Present | 98 | 208 | 260 | 566 |  | | 87 | 205 | 290 | 582 |  |  |
| Absent | 51 | 71 | 126 | 248 |  | | 41 | 76 | 114 | 231 |  |  |
| PND = proximal non-diffuse modified Lauren type; D = diffuse modified Lauren type; DND = distal non-diffuse modified Lauren type; M = male; F = female; | | | | | | | | | | | | |
