# Supplementary material for: Superiority of Tumor Location-Modified Lauren Classification System for Gastric Cancer: A Multi-Institutional Validation Analysis
Source: Ann Surg Oncol. 2018 Jul 26;25(11):3257–63. doi: 10.1245/s10434-018-6654-8 (PMC6132412; doi:10.1245/s10434-018-6654-8)
Supplement: Supplementary file 2 — Supplementary material 2 (DOCX 20 kb) [file 10434_2018_6654_MOESM2_ESM.docx]

| **Table s2. Logistic regression analysis of the risk factors for tumor mLC.** | | | | | |
| --- | --- | --- | --- | --- | --- |
| Factors | Univariate analysis | |  | Multivariate analysis | |
|  | OR(95%CI) | P value |  | OR(95%CI) | P value |
| Gender | 0.821(0.623-0.908) | 0.043 | 0.813(0.690-1.132) | | 0.124 |
| Age | 1.139(1.001-1.575) | 0.032 | 1.079(0.990-1.213) | | 0.436 |
| Macroscopic type | 1.170(0.918-1.809) | 0.123 | － | | － |
| Histologic type | 1.287(1.039-1.953) | <0.001 | 1.032(1.042-1.449) | | 0.033 |
| Tumor size | 1.215(0.882-1.422) | 0.091 | － | | － |
| T stage | 1.107(1.025-1.379) | 0.012 | 1.065(0.922-1.454) | | 0.122 |
| N Stage | 1.534(1.235-2.651) | 0.004 | 1.256(0.957-1.678) | | 0.061 |
| M Stage | 1.702(1.273-2.256) | <0.001 | 1.305(1.202-1.616) | | 0.021 |
| OR=Odds Ratio; CI= Confidence Interval. | | | | | |
