# Supplementary material for: Superiority of Tumor Location-Modified Lauren Classification System for Gastric Cancer: A Multi-Institutional Validation Analysis
Source: Ann Surg Oncol. 2018 Jul 26;25(11):3257–63. doi: 10.1245/s10434-018-6654-8 (PMC6132412; doi:10.1245/s10434-018-6654-8)
Supplement: Supplementary file 3 — Supplementary material 3 (DOCX 24 kb) [file 10434_2018_6654_MOESM3_ESM.docx]

| Table s3. Prognostic prediction by Kaplan-Meier and Life Table analyses | | | | | | | |
| --- | --- | --- | --- | --- | --- | --- | --- |
| Factors | 3-y OS(%) | 5-y OS(%) | MS (95%CI) | AIC | C-index | Log rank χ^2^ value | P |

| LC system |  |  |  | 1012.6 | 0.658 | 114.5 | <0.001 |
| --- | --- | --- | --- | --- | --- | --- | --- |
| Diffuse | 51.2 | 41.9 | 54.9(49.9-57.0) |  |  |  |  |
| Intestinal | 69.3 | 57.1 | 63.0(59.3-67.7) |  |  |  |  |
| Mixed | 63.1 | 51.5 | 61.3(55.4-67.4) |  |  |  |  |
| mLC system |  |  |  | 900.1 | 0.696 | 138.3 | <0.001 |
| D | 51.2 | 41.9 | 54.9(49.9-57.0) |  |  |  |  |
| PND | 60.7 | 53.8 | 62.3(57.3-68.0) |  |  |  |  |
| DND | 67.0 | 54.2 | 64.1(60.9-68.7) |  |  |  |  |
| TNM system |  |  |  | 868.4 | 0.721 | 152.1 | <0.001 |
| I | 90.2 | 87.0 | 85.2(81.3-87.2) |  |  |  |  |
| II | 81.3 | 72.2 | 76.4(71.4-81.1) |  |  |  |  |
| III | 44.1 | 31.6 | 47.3(43.3-50.5) |  |  |  |  |
| IV | 20.4 | 10.9 | 26.2(22.3-30.2) |  |  |  |  |
| Abbreviations: LC=Lauren classification; mLC= modified Lauren classification; D= diffuse type；  PND= proximal non-diffuse type; DND=distal non-diffuse type; OS= overall survival; MS= median survival time;  AIC= Akaike Information Criterion value; C-index= concordance index. | | | | | | | |
