# Supplementary material for: Superiority of Tumor Location-Modified Lauren Classification System for Gastric Cancer: A Multi-Institutional Validation Analysis
Source: Ann Surg Oncol. 2018 Jul 26;25(11):3257–63. doi: 10.1245/s10434-018-6654-8 (PMC6132412; doi:10.1245/s10434-018-6654-8)
Supplement: Supplementary file 4 — Supplementary material 4 (DOCX 49 kb) [file 10434_2018_6654_MOESM4_ESM.docx]

| Table s4. Cox regression analyses predicting overall survival | | | | | |
| --- | --- | --- | --- | --- | --- |
| Factors | Univariate analysis | |  | Multivariate analysis | |
|  | HR(95%CI) | P value |  | HR(95%CI) | P value |
| Gender | 0.854(0.643-0.901) | 0.038 | 0.874(0.662-1.124) | | 0.323 |
| Age (years) | 0.874(0.613-0.989) | 0.017 | 0.943(0.783-1.015) | | 0.089 |
| Macroscopic type | 1.142(1.030-1.473) | 0.016 | 1.022 (0.992-1.349) | | 0.055 |
| Histologic type | 1.211(1.002-1.643) | 0.027 | 1.014(0.893-1.342) | | 0.087 |
| Tumor size | 1.033(1.001-1.246) | 0.043 | 1.015(0.887-1.254) | | 0.091 |
| LC system | 1.212(1.009-1.435) | 0.042 | 1.028(0.929-1.301) | | 0.082 |
| mLC system | 1.412(1.021-1.742) | 0.011 | 1.116(1.018-1.435) | | 0.039 |
| T Stage | 1.947(1.424-2.612) | <0.001 | 1.404(1.091-1.897) | | 0.012 |
| N Stage | 1.723(1.138-2.431) | <0.001 | 1.612(1.127-1.991) | | <0.001 |
| M Stage | 2.226(1.698-2.981) | <0.001 | 1.917(1.622-2.361) | | <0.001 |
| Chemotherapy | 1.556(1.012-2.161) | 0.029 | 1.212(0.949-1.532) | | 0.056 |
| LC=Lauren Classification; mLC=modified Lauren Classification;  HR= Hazard Ratio; CI: =Confidence Interval; | | | | | |
